# Expression and clinical significance of HSP27 and its phosphorylation in lower extremity arteriosclerosis obliterans

**DOI:** 10.7717/peerj.9305

**Published:** 2020-06-11

**Authors:** Junqing Bai, Fule Wang, Xuguang Wang, Eerde Mutu, Chen Duan, Yili Qi, Liqiang Hu, Zhanfeng Gao

**Affiliations:** Department of Vascular Surgery, Affiliated Hospital of Inner Mongolia Medical University, Hohhot, China

**Keywords:** Lower extremity arteriosclerosis obliterans, Heat shock protein 27

## Abstract

**Objectives:**

This study set out to analyze the difference of heat shock protein 27 (HSP27) and its phosphorylation in patients with lower extremity arteriosclerosis obliterans (LEASO) at different stages. This research also examined their clinical significance in this disease.

**Methods:**

Blood samples from 60 patients with LEASO were collected and divided into two groups according to ankle-brachial index (ABI): group A (ABI ≤ 0.43) and group B (ABI > 0.43). The expression of HSP27 in each stage of Fontaine was measured by ELISA, and the difference of HSP27 concentration and ABI between the two groups was analyzed. Meanwhile, three normal femoral artery specimens (normal group) and three atherosclerotic femoral artery specimens (lesion group) were collected, and HSP27 and its Phospho-HSP27 (Ser15), Phospho-HSP27 (Ser78) and Phospho-HSP27 (Ser82) were detected by western blotting. The data of the protein level between the normal group and the lesion group was made a statistical analysis.

**Results:**

HSP27 concentration in group A was (40.73 ± 15.99) ng/ml, and ABI was 0.26 ± 0.20. HSP27 concentration in group B was (66.30 ± 24.70) ng/ml, and ABI was 0.64 ± 0.20. The protein expression of HSP27 and its phosphorylation in the normal group was 0.82 ± 0.13, 0.66 ± 0.12, 0.91 ± 0.24 and 0.90 ± 0.16, respectively; the protein expression of the lesion group was 0.45 ± 0.08, 0.42 ± 0.09, 0.39 ± 0.12 and 0.58 ± 0.11.

**Conclusion:**

Patients with higher LEASO Fontaine stage and lower ABI had a lower HSP27 concentration. Serum HSP27 concentration was negatively correlated with the severity of LEASO, while HSP27 concentration was positively correlated with ABI value. The content of HSP27 and its phosphorylation of lesion group is significantly lower than that of normal group, which may be closely related to the occurrence and development of atherosclerosis.

## Introduction

Atherosclerosis is the main cardiovascular disease leading to ischemia of heart, brain and extremities. Severe cases can lead to infarction (Li et al., 2019). Atherosclerosis has become the first killer threatening human health and the main cause of human death in the world (Jakic et al., 2019). Lower extremity arteriosclerosis obliterans (LEASO) is a chronic progressive disease, in which atherosclerosis causes thickening of the intima of lower extremity arteries, narrowing or even occlusion of the lumen, and further causes a series of symptoms and signs of the affected limb, such as intermittent claudication, resting pain and even ulcer necrosis (Gao et al., 2010). It was reported that the proportion of LEASO complicated with coronary artery disease was significantly higher than that of other diseases, as high as 70% (Aoshima et al., 1992). Atherosclerosis, especially LEASO, is a complex inflammatory disease process that is the major underlying cause of coronary artery disease (Seibert et al., 2013). Current studies on the pathogenesis of atherosclerotic occlusion of the lower limbs indicate that endothelial dysfunction is an early event of atherosclerosis which is significantly related to the progression of atherosclerotic plaques.^8^

Heat shock protein 27 (HSP27), a member of the small HSP family, was identified as a protein with an expression pattern in human coronary arteries that inversely correlated with plaque burden (Al-Madhoun et al., 2007; Miller et al., 2005). HSP is an important stress protective protein in the body, widely distributed in various tissues and cells, and rapidly expressed in large quantities after being stimulated by heat, oxidative stress, or poisons (PulakazhiVenu et al., 2017). HSPs can be divided into HSP100, HSP90, HSP70, HSP60 and the family of small heat shock proteins according to the molecular weight (Chen et al., 2019). Small HSP includes HSP20, HSP22 and HSP27. Phosphorylated heat shock protein 27 (Phospho-HSP27) is an activated form of HSP27.

The expression of HSP27 may decrease in the course of atherosclerosis, and the culture of normal arteries and atherosclerotic plaques shows that the content of HSP27 in normal arteries is higher (Martin-Ventura et al., 2006). By comparing the protein difference of two-dimensional electrophoresis map between normal and arteriosclerosis occlusion, the results showed that the expression of HSP27 from LEASO femoral artery group was obviously lower than that of normal femoral artery. Martín-Ventura et al. (2004) also found that the expression of HSP27 in the complex atherosclerotic plaques was lower and that the level of serum HSP27 in the patients with atherosclerosis was significantly lower than that of healthy controls, which indicated that the level of serum HSP27 may be an indicator for assessing the severity of atherosclerosis. Moreover, a differential proteomics study showed that HSP27 of unstable plaques was significantly reduced compared with that of stable plaques, suggesting that HSP27 plays an important functional role in inflammation and oxidative stress, which may be related to plaque stability (Lepedda et al., 2009). Higher HSP27 levels can predict heart attack, stroke or reduce the risk of cardiovascular disease death. In addition, increasing HSP27 content can weaken experimental atherosclerosis, reduce inflammation, and reduce cholesterol levels (Shi et al., 2019). For atherosclerosis, plasma HSP27 levels could be a potential index of atherosclerosis (Martín-Ventura et al., 2004). Seibert reported that serum HSP27 levels may represent a potential therapeutic target for atherosclerosis (Seibert et al., 2013). However, the expression difference of HSP27 and its phosphorylation sites at different stages of the disease and its role was unclear currently. The aim of this study was to further elaborate on the expression differences of HSP27 and its phosphorylation for LEASO patients of different types and stages. In addition, this study sought to explore its role in LEASO.

## Materials and Methods

### The clinical data of patients and grouping

From September 2017 to December 2018, the clinical data of our hospital was as follows: 60 patients with LEASO, 3 patients with amputation due to the disease and 3 patients with traumatic amputation. All participants gave verbal informed consent before the commencement of the study. The study was approved by the Ethics Committee of The Affiliated Hospital of Inner Mongolia Medical University (approval number: 05083).

The 60 patients with LEASO were not treated by surgery for this disease, and their fasting blood samples were collected. In the Fontaine in installment, 60 patients with LEASO respectively: I period, asymptomatic, 3 cases; II a period, mild intermittent claudication, 16 cases; II b period, moderate to severe intermittent claudication, 13 cases; III period, ischemic resting in 25 cases; IV period, 3 cases of ulcer and gangrene. According to the ankle-brachial index (ABI), 60 patients were divided into two groups: group A (ABI ≤ 0.43) and group B (ABI > 0.43), with 30 cases in each group. Patients from group A were aged from 40 to 65 years old, including 26 males and 4 females, and patients from group B were aged from 41 to 67 years old, including 25 males and 5 females. Three of the six amputees were trauma patients (normal group) and three were atherosclerosis patients (lesion group). Femoral artery samples were collected. In the normal group, there were 2 males and 1 female aged 38 ∼65 who could not retain the affected limb due to trauma. In the lesion group, there were 2 males and 1 female aged 43∼70 who were gangrenous and surgical treatment was ineffective. Due to the anti-atherosclerosis effect of estrogen in females, the female incidence of LEASO is significantly less than male, but the incidence of this disease increases after menopause. General information of group A and B and normal and lesion groups were comparable.

### Enzyme linked immunosorbent assay (ELISA)

Fast blood samples were harvested from our hospital and maintained at 4 °C for 8–12 h. After that, the serum was separated from blood samples and kept in −20 °C for further study. HSP27 of blood samples from 60 patients with LEASO was detected by ELISA kits (Creative Diagnostics NY, USA) according to the manufacturers’ protocol.

### Western blotting

The total protein was separated from blood samples by adding protease inhibitor cocktail (Sigma). Protein extractions were separated by SDS-PAGE and transfected to a PVDF membrane (Millipore). The membrane was blocked with 5% (w/v) reagent-grade nonfat milk (Cell Signaling Technology) and incubated with primary antibodies, including HSP27(ab12351), Phospho-HSP27 (Ser15) (CST, 2404T), Phospho-HSP27 (Ser78) (CST, 2405) and Phospho-HSP27 (Ser82) (CST 2401), at 4 °C overnight followed by secondary antibody incubation. The protein bands were visualized using CLARITYTM Western ECL substrate (Bio-Rad). The protein level was quantified using Image J software normalized with β-actin.

### Observation index

The correlation analysis was performed between HSP27 concentration and ABI and Fontaine stages. Moreover, the correlation analysis was also carried out between the expression of HSP27 and PhosphoHSP27 (Ser15), PhosphoHSP27 (Ser78) and PhosphoHSP27 (Ser82) in 3 normal femoral artery specimens (normal group) and 3 atherosclerotic femoral artery specimens (lesion group).

### Statistical analysis

SPSS 21.0 software was used for statistical analysis. Measurement data were reported as mean ± standard deviation ($\bar {x}$ ± s), and *t*-test was carried out for comparison between groups. *P* < 0.05 was considered to be statistically significant.

## Results

### Correlation between Fontaine each stage and ABI and HSP27 concentration

As shown in Table 1, there was a negative correlation between Fontaine stage and HSP27 concentration or ABI among the 60 LEASO patients. In addition, by analyzing the relationship between HSP27 concentration and different ABI, the results indicated that HSP27 concentration and ABI from group B were significantly higher than those from group A (*P* < 0.05) (Table 2).

**Table 1:** Relationship between Fontaine each stage and ABI and HSP27 concentration.

Fontaine stages	I period	II a period	II b period	III period	IV period
Number of cases (%)	3 (5.00)	16 (26.67)	13 (21.67)	25 (41.67)	3 (5.00)
ABI	0.85 ± 0.05	0.70 ± 0.12	0.49 ± 0.11	0.27 ± 0.16	0.06 ± 0.03
HSP27 concentration (ng/mL)	92.14 ± 5.61	68.55 ± 18.19	55.31 ± 4.68	41.74 ± 12.09	25.00 ± 4.39

**Table 2:** Comparison of HSP27 concentration and ABI between group A and group B ($\bar {x}\pm $s ).

Group	Number of cases	HSP27 concentration (ng/mL)	ABI
Group A	30	40.73 ± 15.99	0.23 ± 0.20
Group B	30	66.30 ± 24.70	0.64 ± 0.20
T value		9.33	14.65
*P* value		<0.05	<0.05

### The relationship between HSP27 concentration and ABI

**Figure 1:**
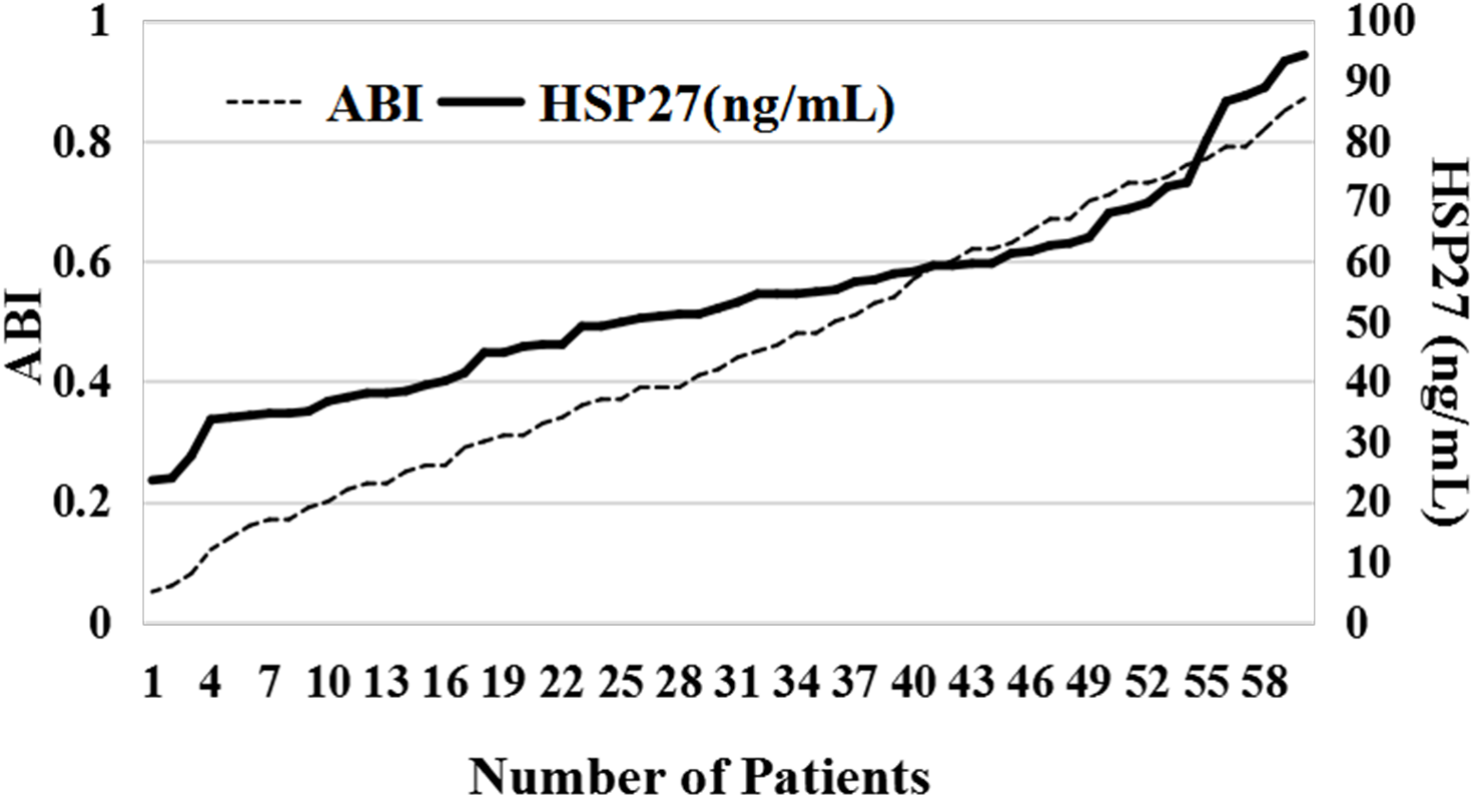
The relationship between HSP27 concentration and ABI.

In Fig. 1, it was found that the higher the concentration of HSP27, the higher the corresponding ABI. With the combination of Tables 1 and 2, ABI was detected to be negatively correlated to the severity of the disease while HSP concentration was positively correlated to the severity of the disease when there was little difference in the age and sex ratio of patients.

### The difference between the protein expression of HSP27 and its three phosphorylation sites from normal and lesion groups

**Figure 2:**
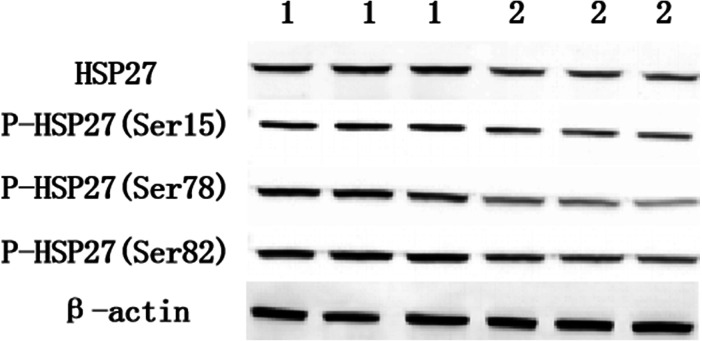
The relative expression of HSP27 and its phosphorylation in normal and lesion groups (1 is normalgroup, 2 is lesion group).

**Figure 3:**
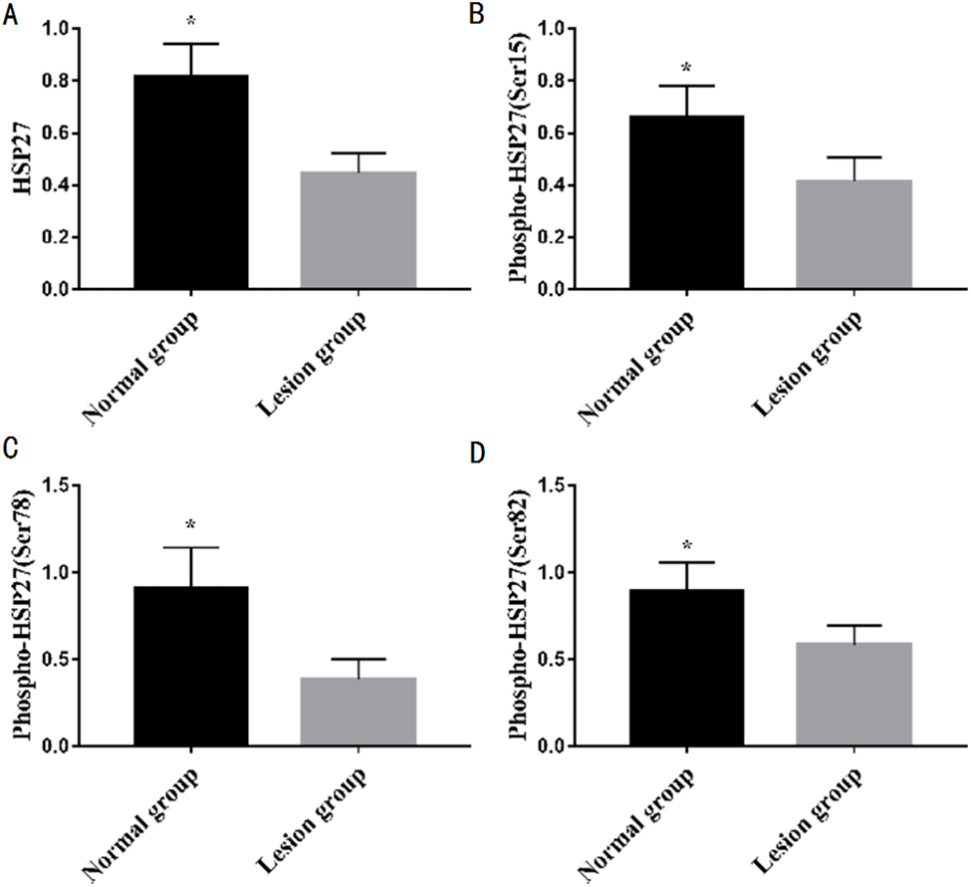
Differences in protein expression of a single indicators between normal and lesion groups. (A) HPS27, (B) Phospho-HSP27 (Ser-15), (C) Phospho-HSP27 (Ser-78) (D) Phospho-HSP27 (Ser-78)

The protein expression levels of HSP27 and its three phosphorylation sites from the normal group and lesion group are shown in Table 3 and Fig. 2. The difference of protein expression for a single indicator is shown in Fig. 3, which is measured multiple times on one respresentive western blot. These results indicated that the protein expression levels of HSP27 and its phosphorylation of the lesion group were significantly lower than those of the normal group (*P* < 0.05).

**Table 3:** Comparison of relative expression of HSP27 and its phosphorylation in normal and lesion groups ($\bar {x}\pm $s ).

Group	HSP27	Phospho-HSP27(Ser15)	Phospho-HSP27(Ser78)	Phospho-HSP27(Ser82)
Normal group	0.82 ± 0.13	0.66 ± 0.12	0.91 ± 0.24	0.90 ± 0.16
Lesion group	0.45 ± 0.08	0.42 ± 0.09	0.39 ± 0.12	0.58 ± 0.11
T value	4.36	2.90	3.40	2.79
*P* value	0.01	0.04	0.03	0.04

## Discussion

**Figure 4:**
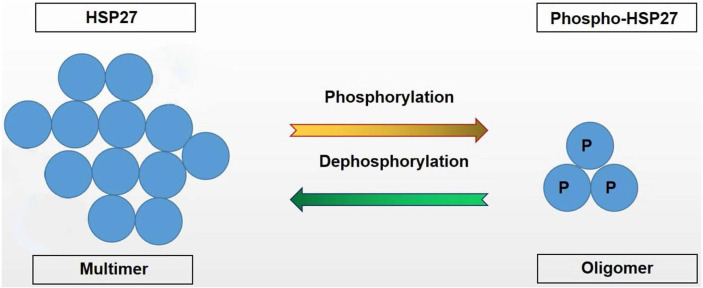
HSP27 and Phospho-HSP27 structural transformation.

Under physiological conditions, when cells are not under stress, the expression of HSP27 is low and mainly exists as large polymers, which suggest generally inactive and mainly serve as molecular chaperones to regulate the transport, folding and assembly of peptides. When cells are stimulated by biological or physical factors, the expression of HSP27 increases, and the large polymers depolymerized to form oligomers, which is mainly regulated by molecular phosphorylation of HSP27 (Fig. 4);.There are three phosphorylation sites in HSP27: Ser-15, Ser-78 and Ser-82, in which Ser-78 and Ser-82 are located at the C terminal and Ser-15 is located at the N terminal (Salinthone, Tyagi & Gerthoffer, 2008). The latest evidence suggests that phosphorylated HSP27 is an effective anti-apoptotic molecule. In a rat model of aortic atherosclerosis induced by a high cholesterol diet, 46 proteins in the aortic tissue were differentially expressed, and the level of phosphorylated HSP27 was reduced. It has also been reported that phosphorylated HSP27 was abundant in the conditioned medium of normal arterial intima samples, but was rarely found in conditioned medium of atherosclerosis. Myocardial biopsies showed that the phosphorylation of HSP27 from patients diagnosed with atherosclerosis was about 20 times lower than that in normal subjects (Ghayour-Mobarhan, Saber & Ferns, 2012).

However, the correlation between the expression of HSP27 and its phosphorylation level from LEASO patients and Fontaine different stages were specifically analyzed so as to confirm the unclear role of HASP27 in LEASO in China. The results of the present study manifested that the higher the LEASO stage, the lower the ABI, the lower the HSP27 concentration; on the contrary, the lower the LEASO stage, the higher the ABI, the higher the HSP27 concentration (Table 1). HSP27 expression and ABI from lesion group were significantly lower than those of normal group (Table 2). ABI has been used in clinical application for more than 40 years. It is a more accurate screening test for lower extremity arteriosclerosis obliterans. Based on the measured values, the degree and location of lower limb ischemia can be preliminarily judged. The results of previous studies on ABI unanimously agree with the fact that ABI is of great value in the ischemic assessment of LEASO, with high accuracy (Alqahtani et al., 2018). In Fig. 1, the higher the concentration of HSP27, the higher the corresponding ABI, and the lower the concentration of HSP27, the lower the corresponding ABI. Accordingly, it can come to conclusion that the HSP27 concentration is positively correlated with the ABI value. Moreover, the size of ABI reflects the severity of LEASO, that is, the lower the ABI of the patient, the more serious the disease and the lower the concentration of HSP27. When the concentration of HSP27 increases, the progress of the disease slows down, and vice versa. Therefore, HSP27 can be used as a biological index to evaluate atherosclerosis, indicating that serum HSP27 concentration is negatively correlated with the severity of LEASO, and HSP27 concentration is positively correlated with ABI value. Furthermore, it was found that the protein expression of HSP27 and its three phosphorylation sites of the lesion group was significantly lower than that of the normal group (*P* < 0.05) in Table 3. That is to say, the content of HSP27 and its phosphorylation of atherosclerotic patients femoral artery is greatly lower than that of normal human femoral artery. At present, there are few reports on the normal human HSP27 and its phosphorylation content. In this study, the HSP27 concentration and the protein expression of HSP27 from blood samples were determined, and the correlation between HSP27 concentration and ABI or Fontaine stages was analyzed. Although LEASO clinical patients from 2017 to 2018 at our hospital were investigated, the results also would provide good help for the role of HSP27 in LEASO patients in the further studies.

## Conclusion

In summary, serum HSP27 concentration was negatively correlated with the severity of LEASO, while HSP27 concentration was positively correlated with ABI value. The protein expression of HSP27 and its phosphorylation of atherosclerotic patients’ femoral artery is hugely lower than those of normal human femoral artery, which may be closely related to the occurrence and development of LEASO. This will lay the foundation for the future investigation of the diagnosis, pathogenesis and treatment of LEASO.

##  Supplemental Information

10.7717/peerj.9305/supp-1 Supplemental Information 1 Raw data

## Additional Information and Declarations

### Data Availability

The following information was supplied regarding data availability: The raw data is at available at Figshare: Gao (2019): Fig. 1 data. figshare. Dataset. https://doi.org/10.6084/m9.figshare.11445801.v1. Gao (2020): Fig. 2. figshare. Dataset. https://doi.org/10.6084/m9.figshare.11445846.v1. Gao (2020): Fig. 3. figshare. Dataset. https://doi.org/10.6084/m9.figshare.11445855.v1.
